# Difluoridodioxido(1,10-phenanthroline)molybdenum(VI)

**DOI:** 10.1107/S1600536809031626

**Published:** 2009-08-19

**Authors:** Wenju Wang, Youdi Zhang, Xiangjun Jin, Xiguang Du

**Affiliations:** a Department of Chemistry, Baicheng Normal College, Baicheng, Jiln 137000, People’s Republic of China; b College of Chemistry, Northeast Normal University, Changchun, Jilin 130024, People’s Republic of China

## Abstract

The title compound, [MoF_2_O_2_(C_12_H_8_N_2_)], has non-crystallographic mirror symmetry. The Mo^VI^ atom shows a distorted octa­hedral environment, with the phenanthroline N atoms and the two oxide groups forming the equatorial plane and the F atoms occupying the apical positions. Weak C—H⋯O and C—H⋯F hydrogen-bonding contacts and π–π inter­actions [centroid–centroid distance = 3.662 (1) Å] connect the complex mol­ecules into a three-dimensional supra­molecular framework.

## Related literature

For the structure and mode of action of the co-factor of oxido-molybdoenzymes, see: Collison *et al.* (1996 ▶). For the catalyst precursors, see Villata *et al.* (2000 ▶). For the dichlorido­dioxo analogue of the title compound, see: Viossat & Rodier (1979 ▶). For other related structures with the chelating phenanthroline ligand, see: Butcher *et al.* (1979 ▶); Bingham *et al.* (2006 ▶); Zhou *et al.* (2000 ▶).
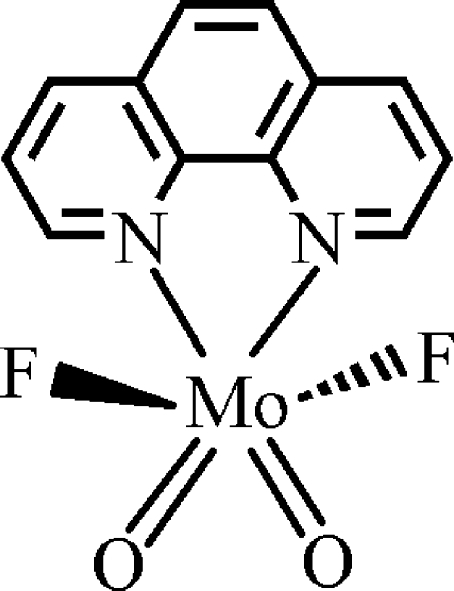


## Experimental

### 

#### Crystal data

[MoF_2_O_2_(C_12_H_8_N_2_)]
*M* *_r_* = 346.14
Monoclinic, 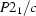

*a* = 7.5190 (9) Å
*b* = 17.818 (2) Å
*c* = 9.5331 (11) Å
β = 110.8560 (10)°
*V* = 1193.5 (2) Å^3^
*Z* = 4
Mo *K*α radiation
μ = 1.12 mm^−1^
*T* = 295 K
0.30 × 0.30 × 0.20 mm

#### Data collection

Bruker APEXII diffractometer
Absorption correction: multi-scan (*SADABS*; Sheldrick, 1996 ▶) *T*_min_ = 0.711, *T*_max_ = 0.799
6460 measured reflections
2394 independent reflections
2248 reflections with *I* > 2σ(*I*)
*R*_int_ = 0.023

#### Refinement

*R*[*F* ^2^ > 2σ(*F* ^2^)] = 0.022
*wR*(*F* ^2^) = 0.065
*S* = 1.04
2394 reflections
173 parameters
H-atom parameters constrained
Δρ_max_ = 0.46 e Å^−3^
Δρ_min_ = −0.59 e Å^−3^

### 

Data collection: *APEX2* (Bruker, 2003 ▶); cell refinement: *SAINT* (Bruker, 2001 ▶); data reduction: *SAINT*; program(s) used to solve structure: *SHELXS97* (Sheldrick, 2008 ▶); program(s) used to refine structure: *SHELXL97* (Sheldrick, 2008 ▶); molecular graphics: *DIAMOND* (Brandenburg & Berndt, 1999 ▶); software used to prepare material for publication: *SHELXL97*.

## Supplementary Material

Crystal structure: contains datablocks I, global. DOI: 10.1107/S1600536809031626/si2192sup1.cif

Structure factors: contains datablocks I. DOI: 10.1107/S1600536809031626/si2192Isup2.hkl

Additional supplementary materials:  crystallographic information; 3D view; checkCIF report

## Figures and Tables

**Table 1:** Selected bond lengths (Å)

Mo1—O2	1.6874 (18)
Mo1—O1	1.6936 (17)
Mo1—F1	1.9017 (14)
Mo1—F2	1.9049 (13)
Mo1—N2	2.3257 (18)
Mo1—N1	2.3295 (18)

**Table 2:** Hydrogen-bond geometry (Å, °)

*D*—H⋯*A*	*D*—H	H⋯*A*	*D*⋯*A*	*D*—H⋯*A*
C2—H2*C*⋯F1^i^	0.93	2.45	3.202 (3)	138
C3—H3*C*⋯O1^ii^	0.93	2.55	3.376 (3)	148
C7—H7⋯F1^iii^	0.93	2.44	3.191 (3)	137
C8—H8⋯O2^iv^	0.93	2.59	3.222 (3)	126

Symmetry codes: (i) 
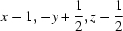
; (ii) 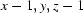; (iii) 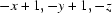; (iv) 
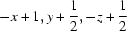
.

**Fig. 1.**
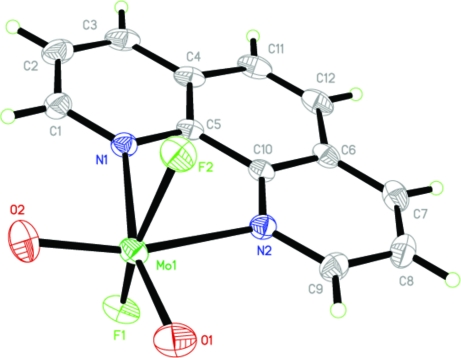
The local coordination environment of the Mo(VI) atom in the title compound drawn with 30% probability. H atoms omitted for clarity.

**Fig. 2.**
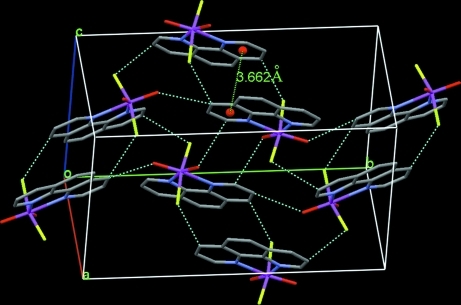
The three-dimensional supramolecular network of the title compund produced by hydrogen-bonding and π–π stacking interactions.[Color codes: Mo, pink; O, red; F, yellow; N, blue; C, grey. Symmetry codes: (i) x - 1, -y + 1/2, z - 1/2; (ii) x - 1, y, z - 1; (iii) -x + 1, -y + 1, -z; (iv) -x + 1, y + 1/2, -z + 1/2.]

## supplementary crystallographic information

### Comment

The high oxidation state oxomolybdenum(VI) can be potentially used as molybdoenzyme, oxo transfer agents, and catalyst precusors [Collison, *et al.*, (1996); Villata, *et al.*, (2000)]. Though some MoO_2_*X*_2_ (*X* = Cl, Br) complexes have been reported [Butcher, *et al.* (1979)], these compounds are usually unstable in air. Thus, the air stable dioxomolybdenum(VI) complex remains an intriguing area for chemists [Bingham, *et al.* (2006)]. In this paper, we reported a new fluorin containing oxomolybdenum(VI) complex that is stable in air.

As shown in Fig. 1, the title complex exhibits non-crystallographic molecular mirror symmetry. The distorted octahedral environment around molybdenum consists of *cis* terminal oxygen atoms (O_t_) and *trans* fluorin atoms and bidentate 1,10-phenanthroline ligand. One mirror plane can be seen bisecting the atoms F1-Mo1-F2 and extending through the midpoints of the central C—C bonds of the phenanthroline ligand, while another mirror can be imagined within the phenanthroline plane and the Mo and dioxo atoms. The average Mo—O_t_ bond distance of 1.691 Å (Table 1) is a typical molybdenum-oxygen terminal double bond and is similar to those observed in MoO_2_*X*_2_.2*L* complexes [Butcher, *et al.* (1979)]. The Mo—N bond distances (2.326 (2) Å and 2.330 (2) Å) are also similar to those values observed in analogue complexes [Bingham, *et al.* (2006); Viossat & Rodier, (1979)]. However, the Mo—F bond distances of 1.905 (1) Å and 1.902 (1) Å for the title compound are transparently shorter than the Mo—Cl or Mo—Br bonds determined in other MoO_2_*X*_2_ (*X* = Cl, Br) complexes [For MoO_2_*Cl*_2_ complex, see: Viossat & Rodier, (1979); for MoO_2_*X*_2_.2*L* complexes, see: Butcher, *et al.* (1979)]. This is the main reason that the title compound is stable in air. Furthermore, there also exist weak C—H···F and C—H···O hydrogen bonding interactions between neighboring molecules which plays an important role to consolidate the supramolecular structure of the title compund. The detailed hydrogen bond parameters are shown in Table 2. Molybdenum and 1,10-phenanthroline complexes were substantively reported [Zhou,*et al.* (2000); Viossat & Rodier, (1979); Butcher, *et al.* (1979)], but it was quite missing that some references described the distinctive nature or features of *π*–*π* interaction. Whereas for the title complex, the 1,10-phenanthroline phenyl ring induced *π*–*π* interaction is demonstrated to aid the three-dimensional structure together with the weak hydrogen bonding contacts (Fig. 2). The centroid to centroid distance is 3.6619 (14) Å. (Cg3···Cg3îii^, Cg3 is the centroid of the ring (N2 C9 C8 C7 C6 C10), symmetry code iii = 1 - *x*, 1 - *y*, -*z*). The perpendicular distance of the rings is 3.369 Å and the slippage between the rings is 1.435 Å.

### Experimental

A mixture of Molybdenum trioxide (0.2874 g, 2 mmol), HF (2 ml), 1,10-phenanthroline(0.2246 g, 1.1 mmol) and *N*,*N*-dimethylformamide (30 ml) was stirring for 7 h under 343 K. After cooling to room temperature, the mixture was adjusted to pH = 6.05 with 6 *M* H_2_SO_4_ solution. The filtration was allowed to stand over several days to give colorless block single crystals in 80% yield. Analysis calculated for C_12_H_8_F_2_MoN_2_O_2_: C 41.64, H 2.33, N 8.09, F 10.98%; found: C 41.60, H 2.30, N 8.06, F 10.94%.

### Refinement

H atoms were located from difference Fourier maps, but were subsequently placed in calculated positions and treated as riding, with C—H = 0.93 Å. All H atoms were allocated displacement parameters related to those of their parent atoms [*U*_iso_(H) = 1.2 *U*eq (C,*O*)]

### Crystal data

**Table:**

[MoF_2_O_2_(C_12_H_8_N_2_)]	*F*(000) = 680
*M**_r_* = 346.14	*D*_x_ = 1.926 Mg m^−^^3^
Monoclinic, *P*2_1_/*c*	Mo *K*α radiation, λ = 0.71073 Å
Hall symbol: -P 2ybc	Cell parameters from 25 reflections
*a* = 7.5190 (9) Å	θ = 7.5–15°
*b* = 17.818 (2) Å	µ = 1.12 mm^−^^1^
*c* = 9.5331 (11) Å	*T* = 295 K
β = 110.856 (1)°	Block, colorless
*V* = 1193.5 (2) Å^3^	0.30 × 0.30 × 0.20 mm
*Z* = 4	

### Data collection

**Table:**

Bruker APEXII diffractometer	2248 reflections with *I* > 2σ(*I*)
Radiation source: fine-focus sealed tube	*R*_int_ = 0.023
graphite	θ_max_ = 26.2°, θ_min_ = 2.6°
ω scans	*h* = −9→8
Absorption correction: multi-scan (*SADABS*; Sheldrick, 1996)	*k* = −15→22
*T*_min_ = 0.711, *T*_max_ = 0.799	*l* = −11→11
6460 measured reflections	2 standard reflections every 150 reflections
2394 independent reflections	intensity decay: none

### Refinement

**Table:**

Refinement on *F*^2^	Secondary atom site location: difference Fourier map
Least-squares matrix: full	Hydrogen site location: inferred from neighbouring sites
*R*[*F*^2^ > 2σ(*F*^2^)] = 0.022	H-atom parameters constrained
*wR*(*F*^2^) = 0.065	*w* = 1/[σ^2^(*F*_o_^2^) + (0.04*P*)^2^ + 0.45*P*] where *P* = (*F*_o_^2^ + 2*F*_c_^2^)/3
*S* = 1.03	(Δ/σ)_max_ = 0.001
2394 reflections	Δρ_max_ = 0.46 e Å^−^^3^
173 parameters	Δρ_min_ = −0.59 e Å^−^^3^
0 restraints	Extinction correction: *SHELXL97* (Sheldrick, 2008), Fc^*^=kFc[1+0.001xFc^2^λ^3^/sin(2θ)]^-1/4^
0 constraints	Extinction coefficient: 0.0345 (14)
Primary atom site location: structure-invariant direct methods	

### Special details

**Table:**

Geometry. All e.s.d.'s (except the e.s.d. in the dihedral angle between two l.s. planes) are estimated using the full covariance matrix. The cell e.s.d.'s are taken into account individually in the estimation of e.s.d.'s in distances, angles and torsion angles; correlations between e.s.d.'s in cell parameters are only used when they are defined by crystal symmetry. An approximate (isotropic) treatment of cell e.s.d.'s is used for estimating e.s.d.'s involving l.s. planes.
Refinement. Refinement of *F*^2^ against ALL reflections. The weighted *R*-factor *wR* and goodness of fit *S* are based on *F*^2^, conventional *R*-factors *R* are based on *F*, with *F* set to zero for negative *F*^2^. The threshold expression of *F*^2^ > σ(*F*^2^) is used only for calculating *R*-factors(gt) *etc*. and is not relevant to the choice of reflections for refinement. *R*-factors based on *F*^2^ are statistically about twice as large as those based on *F*, and *R*- factors based on ALL data will be even larger.

### Fractional atomic coordinates and isotropic or equivalent isotropic displacement parameters (Å^2^)

**Table:**

	*x*	*y*	*z*	*U*_iso_*/*U*_eq_	
Mo1	0.46789 (2)	0.344747 (10)	0.354935 (18)	0.03131 (11)	
F1	0.62030 (19)	0.32745 (9)	0.23609 (16)	0.0466 (3)	
F2	0.25657 (19)	0.38491 (8)	0.39576 (15)	0.0441 (3)	
O1	0.6429 (3)	0.38223 (12)	0.50412 (19)	0.0548 (5)	
O2	0.4418 (3)	0.25454 (10)	0.3980 (2)	0.0531 (5)	
N1	0.2347 (2)	0.32975 (10)	0.1180 (2)	0.0305 (4)	
N2	0.4234 (2)	0.45752 (10)	0.22405 (18)	0.0299 (4)	
C1	0.1412 (3)	0.26638 (13)	0.0684 (3)	0.0397 (5)	
H1	0.1689	0.2247	0.1313	0.048*	
C2	0.0030 (3)	0.25974 (15)	−0.0746 (3)	0.0477 (6)	
H2C	−0.0610	0.2145	−0.1052	0.057*	
C3	−0.0379 (3)	0.31953 (18)	−0.1689 (3)	0.0462 (6)	
H3C	−0.1304	0.3155	−0.2643	0.055*	
C4	0.0602 (3)	0.38758 (14)	−0.1221 (2)	0.0365 (5)	
C5	0.1962 (3)	0.38964 (12)	0.0244 (2)	0.0282 (4)	
C6	0.2597 (3)	0.52244 (13)	−0.0100 (2)	0.0343 (4)	
C7	0.3596 (3)	0.58803 (13)	0.0530 (3)	0.0417 (5)	
H7	0.3414	0.6317	−0.0037	0.050*	
C8	0.4833 (4)	0.58742 (13)	0.1978 (3)	0.0442 (5)	
H8	0.5477	0.6309	0.2414	0.053*	
C9	0.5125 (3)	0.52088 (13)	0.2799 (3)	0.0376 (5)	
H9	0.5982	0.5210	0.3783	0.045*	
C10	0.2964 (3)	0.45787 (11)	0.0808 (2)	0.0281 (4)	
C11	0.0272 (3)	0.45398 (16)	−0.2121 (2)	0.0456 (6)	
H11	−0.0617	0.4529	−0.3093	0.055*	
C12	0.1218 (3)	0.51780 (15)	−0.1590 (3)	0.0443 (6)	
H12	0.0974	0.5600	−0.2204	0.053*	

### Atomic displacement parameters (Å^2^)

**Table:**

	*U*^11^	*U*^22^	*U*^33^	*U*^12^	*U*^13^	*U*^23^
Mo1	0.03177 (14)	0.03595 (15)	0.02202 (14)	0.00454 (6)	0.00442 (9)	0.00358 (6)
F1	0.0367 (7)	0.0648 (9)	0.0385 (7)	0.0141 (6)	0.0137 (6)	0.0061 (7)
F2	0.0462 (7)	0.0492 (8)	0.0420 (7)	0.0054 (6)	0.0220 (6)	−0.0004 (6)
O1	0.0492 (10)	0.0685 (13)	0.0312 (9)	−0.0001 (9)	−0.0045 (8)	−0.0006 (8)
O2	0.0691 (12)	0.0409 (10)	0.0500 (10)	0.0100 (8)	0.0223 (9)	0.0131 (8)
N1	0.0280 (8)	0.0330 (9)	0.0283 (9)	0.0003 (7)	0.0073 (7)	−0.0023 (7)
N2	0.0287 (8)	0.0329 (9)	0.0256 (8)	−0.0001 (7)	0.0067 (7)	−0.0006 (7)
C1	0.0349 (11)	0.0369 (12)	0.0453 (13)	−0.0036 (9)	0.0116 (10)	−0.0063 (10)
C2	0.0335 (11)	0.0517 (15)	0.0535 (15)	−0.0088 (10)	0.0102 (11)	−0.0207 (12)
C3	0.0266 (11)	0.0688 (17)	0.0355 (12)	0.0024 (10)	0.0016 (9)	−0.0172 (12)
C4	0.0240 (9)	0.0558 (14)	0.0265 (10)	0.0064 (9)	0.0052 (8)	−0.0044 (9)
C5	0.0230 (9)	0.0381 (11)	0.0223 (9)	0.0047 (7)	0.0067 (7)	−0.0017 (8)
C6	0.0332 (10)	0.0396 (11)	0.0349 (11)	0.0099 (9)	0.0181 (9)	0.0081 (9)
C7	0.0499 (13)	0.0352 (12)	0.0496 (14)	0.0079 (10)	0.0296 (12)	0.0091 (10)
C8	0.0536 (14)	0.0333 (12)	0.0537 (15)	−0.0079 (10)	0.0290 (12)	−0.0064 (10)
C9	0.0375 (11)	0.0396 (12)	0.0341 (11)	−0.0050 (9)	0.0109 (9)	−0.0066 (9)
C10	0.0252 (9)	0.0349 (10)	0.0247 (9)	0.0060 (7)	0.0097 (7)	0.0013 (8)
C11	0.0335 (11)	0.0714 (17)	0.0265 (11)	0.0142 (11)	0.0042 (9)	0.0071 (11)
C12	0.0404 (12)	0.0587 (15)	0.0346 (11)	0.0186 (11)	0.0142 (10)	0.0183 (11)

### Geometric parameters (Å, °)

**Table:**

Mo1—O2	1.6874 (18)	C3—H3C	0.9300
Mo1—O1	1.6936 (17)	C4—C5	1.407 (3)
Mo1—F1	1.9017 (14)	C4—C11	1.430 (4)
Mo1—F2	1.9049 (13)	C5—C10	1.431 (3)
Mo1—N2	2.3257 (18)	C6—C7	1.403 (3)
Mo1—N1	2.3295 (18)	C6—C10	1.407 (3)
N1—C1	1.325 (3)	C6—C12	1.432 (3)
N1—C5	1.355 (3)	C7—C8	1.362 (4)
N2—C9	1.324 (3)	C7—H7	0.9300
N2—C10	1.360 (2)	C8—C9	1.395 (3)
C1—C2	1.394 (3)	C8—H8	0.9300
C1—H1	0.9300	C9—H9	0.9300
C2—C3	1.357 (4)	C11—C12	1.341 (4)
C2—H2C	0.9300	C11—H11	0.9300
C3—C4	1.407 (4)	C12—H12	0.9300
			
O2—Mo1—O1	107.12 (10)	C4—C3—H3C	120.1
O2—Mo1—F1	97.89 (8)	C3—C4—C5	116.8 (2)
O1—Mo1—F1	96.38 (8)	C3—C4—C11	124.3 (2)
O2—Mo1—F2	97.50 (8)	C5—C4—C11	118.9 (2)
O1—Mo1—F2	97.80 (8)	N1—C5—C4	122.9 (2)
F1—Mo1—F2	154.96 (6)	N1—C5—C10	117.49 (17)
O2—Mo1—N2	161.15 (8)	C4—C5—C10	119.63 (19)
O1—Mo1—N2	91.73 (8)	C7—C6—C10	117.4 (2)
F1—Mo1—N2	79.77 (6)	C7—C6—C12	124.1 (2)
F2—Mo1—N2	79.28 (6)	C10—C6—C12	118.5 (2)
O2—Mo1—N1	90.79 (8)	C8—C7—C6	119.7 (2)
O1—Mo1—N1	162.00 (8)	C8—C7—H7	120.1
F1—Mo1—N1	78.99 (6)	C6—C7—H7	120.1
F2—Mo1—N1	81.17 (6)	C7—C8—C9	119.3 (2)
N2—Mo1—N1	70.38 (6)	C7—C8—H8	120.3
C1—N1—C5	118.29 (19)	C9—C8—H8	120.3
C1—N1—Mo1	124.37 (16)	N2—C9—C8	122.9 (2)
C5—N1—Mo1	117.32 (13)	N2—C9—H9	118.6
C9—N2—C10	118.29 (19)	C8—C9—H9	118.6
C9—N2—Mo1	124.43 (14)	N2—C10—C6	122.38 (19)
C10—N2—Mo1	117.28 (13)	N2—C10—C5	117.45 (17)
N1—C1—C2	122.5 (2)	C6—C10—C5	120.16 (18)
N1—C1—H1	118.7	C12—C11—C4	121.3 (2)
C2—C1—H1	118.7	C12—C11—H11	119.3
C3—C2—C1	119.7 (2)	C4—C11—H11	119.3
C3—C2—H2C	120.2	C11—C12—C6	121.5 (2)
C1—C2—H2C	120.2	C11—C12—H12	119.2
C2—C3—C4	119.8 (2)	C6—C12—H12	119.2
C2—C3—H3C	120.1		
			
O2—Mo1—N1—C1	0.08 (18)	Mo1—N1—C5—C10	−2.4 (2)
O1—Mo1—N1—C1	174.5 (3)	C3—C4—C5—N1	0.4 (3)
F1—Mo1—N1—C1	97.96 (18)	C11—C4—C5—N1	179.07 (19)
F2—Mo1—N1—C1	−97.39 (17)	C3—C4—C5—C10	−177.92 (18)
N2—Mo1—N1—C1	−179.08 (18)	C11—C4—C5—C10	0.8 (3)
O2—Mo1—N1—C5	−178.35 (15)	C10—C6—C7—C8	−1.2 (3)
O1—Mo1—N1—C5	−3.9 (3)	C12—C6—C7—C8	177.7 (2)
F1—Mo1—N1—C5	−80.46 (14)	C6—C7—C8—C9	1.6 (3)
F2—Mo1—N1—C5	84.18 (14)	C10—N2—C9—C8	−0.8 (3)
N2—Mo1—N1—C5	2.49 (13)	Mo1—N2—C9—C8	179.51 (16)
O2—Mo1—N2—C9	174.7 (2)	C7—C8—C9—N2	−0.6 (3)
O1—Mo1—N2—C9	−4.66 (18)	C9—N2—C10—C6	1.3 (3)
F1—Mo1—N2—C9	−100.83 (17)	Mo1—N2—C10—C6	−179.05 (14)
F2—Mo1—N2—C9	92.95 (17)	C9—N2—C10—C5	−177.63 (18)
N1—Mo1—N2—C9	177.31 (18)	Mo1—N2—C10—C5	2.1 (2)
O2—Mo1—N2—C10	−5.0 (3)	C7—C6—C10—N2	−0.3 (3)
O1—Mo1—N2—C10	175.66 (15)	C12—C6—C10—N2	−179.26 (19)
F1—Mo1—N2—C10	79.49 (14)	C7—C6—C10—C5	178.60 (17)
F2—Mo1—N2—C10	−86.72 (14)	C12—C6—C10—C5	−0.4 (3)
N1—Mo1—N2—C10	−2.37 (13)	N1—C5—C10—N2	0.2 (3)
C5—N1—C1—C2	−1.4 (3)	C4—C5—C10—N2	178.62 (17)
Mo1—N1—C1—C2	−179.78 (17)	N1—C5—C10—C6	−178.69 (17)
N1—C1—C2—C3	0.9 (4)	C4—C5—C10—C6	−0.3 (3)
C1—C2—C3—C4	0.3 (4)	C3—C4—C11—C12	178.0 (2)
C2—C3—C4—C5	−0.9 (3)	C5—C4—C11—C12	−0.6 (3)
C2—C3—C4—C11	−179.5 (2)	C4—C11—C12—C6	−0.2 (3)
C1—N1—C5—C4	0.7 (3)	C7—C6—C12—C11	−178.3 (2)
Mo1—N1—C5—C4	179.25 (14)	C10—C6—C12—C11	0.6 (3)
C1—N1—C5—C10	179.07 (18)		

### Hydrogen-bond geometry (Å, °)

**Table:**

*D*—H···*A*	*D*—H	H···*A*	*D*···*A*	*D*—H···*A*
C2—H2C···F1^i^	0.93	2.45	3.202 (3)	138
C3—H3C···O1^ii^	0.93	2.55	3.376 (3)	148
C7—H7···F1^iii^	0.93	2.44	3.191 (3)	137
C8—H8···O2^iv^	0.93	2.59	3.222 (3)	126

Symmetry codes: (i) *x*−1, −*y*+1/2, *z*−1/2; (ii) *x*−1, *y*, *z*−1; (iii) −*x*+1, −*y*+1, −*z*; (iv) −*x*+1, *y*+1/2, −*z*+1/2.
